# The Influence of Multiple Extrusions on the Properties of High Filled Polylactide/Multiwall Carbon Nanotube Composites

**DOI:** 10.3390/ma15248958

**Published:** 2022-12-15

**Authors:** Daniel Kaczor, Krzysztof Bajer, Aneta Raszkowska-Kaczor, Grzegorz Domek, Piotr Madajski, Pawel Szroeder

**Affiliations:** 1 Łukasiewicz Research Network—Institute for Engineering of Polymer Materials and Dyes, Marii Skłodowskiej-Curie 55, 87-100 Toruń, Poland; 2 Faculty of Mechatronics, Kazimierz Wielki University, Kopernika 1, 85-074 Bydgoszcz, Poland; 3 Faculty of Chemistry, Nicolaus Copernicus University, Gagarina 7, 87-100 Toruń, Poland; 4 Institute of Physics, Kazimierz Wielki University, Powstańców Wielkopolskich 2, 85-090 Bydgoszcz, Poland

**Keywords:** poly(lactic acid), multiwall carbon nanotubes, composites, single-screw extruder, twin-screw extruder, polymer crystallinity, melting flow index, multiple extrusion

## Abstract

High filled polylactide/multiwall carbon nanotube composites were subjected to multiple extrusions using single-screw and twin-screw extruders. Samples of the processed composites were characterized by SEM, XRD, Raman, and FTIR spectroscopy. Thermal and rheological properties were investigated by DSC and MFR analyses. Subsequent extrusions resulted in decreased torque and process efficiency, which is a consequence of the viscosity reduction of PLA. Thermal and rheological properties of composites changed after each extrusion as well. As revealed by DSC analyses, cold crystallization temperature showed a tendency to decrease after each process, whereas cold crystallization enthalpy ΔHcc increased significantly. Melt flow rate, which is indicative of the polymer degradation, increased after each extrusion.

## 1. Introduction

Due to its biodegradable features, polylactide (PLA) has attracted extensive industrial and academic interest as a substitute for petroleum-based polymers [1]. The bottleneck in potential PLA applications on an industrial scale is its impact strength, heat distortion temperature, and gas barrier properties [2]. One way to overcome these limitations is the modification of PLA with fillers to achieve its compatibility with end-use requirements [3,4]. A simple way to improve final properties of PLA is using carbon nanotubes (CNT), which are characterized by a high aspect ratio [5]. CNTs incorporated into the PLA matrix play a reinforcement role that results in better maximum tensile strength [6]. Another effect of the CNT filler is the improved electrical conductivity of the composite [7]. The addition of CNTs results in the improved mechanical and thermal properties of the composite, which can be used in 3D printing [8].

The final properties of PLA/CNT depend on both the filler concentration and dispersion of tubules that should form a network structure in the PLA matrix [9]. The processing conditions have a great influence on the structure of the nanotube network. In particular, CNT concentration affects the distribution and dispersion of CNTs in the PLA in the extrusion process [10]. In this work, we focus on the extrusion process of high filled (up to 25 wt.%) PLA/CNT composites. These types of composites can be used as polymer masterbatches, greatly simplifying the dosing of nanomaterials during extrusion and, thus, obtaining composites with a low concentration of such additives. The blends of PLA and multiwalled carbon nanotubes (MWCNTs) have been extruded one, two, or three times. 

## 2. Materials and Methods

### 2.1. Materials

The polylactide (PLA) produced by Total-Corbion (Gorinchem, The Netherlands), available under the trade name Luminy^®^ LX175 (sample marked as PLA/P), was used as a polymer matrix. According to the product data sheet, PLA is characterized by a density of 1.24 g/cm^3^, stereochemical purity of 96%, residual monomer content of ≤0.3%, melting temperature of 155 °C, and glass transition temperature of 60 °C. As-received material was ground into a powder with a particle size of 100–600 µm. 

Industrial grade multiwall carbon nanotubes (MWCNT), with length 10–30 µm, outside diameter 10–30 nm, and inside diameter 5–10 nm were used as fillers in the obtained composites. 

### 2.2. Composite Preparation

Before the extruding process, PLA was dried in a POL-EKO SLW 180 STD dryer at temperature 80 °C for 8 h. Two series of samples containing 25 wt.% of MWCNT were prepared. 

**Figure 1:**
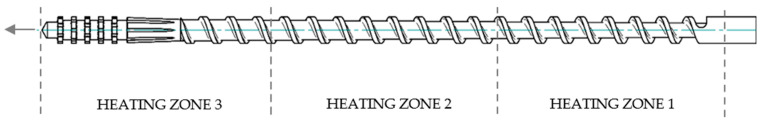
Screw configuration of the single-screw extruder.

The first one was made by using single-screw Brabender Lab-Station extruder (Brabender, Germany), equipped with a screw of 19-m diameter and L/D (length-to-diameter) ratio of 25. To improve mixing abilities, a screw with Maddock shearing section and with a pin mixing section was used (Figure 1). Maddock element was used to rub agglomerates and trap and melt solid polymer particles. All samples were extruded at the screw rotation speed: 50 rpm. Temperatures of the heating zones of the extruder barrel and extruder head were set to 170 °C, 175 °C, 180 °C, and 180 °C, respectively.

**Figure 2:**
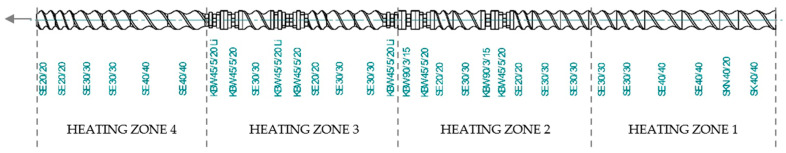
Screw configuration of the co-rotating, twin-screw extruder, where SK—feeding element, SE—conveying element, KBW—kneading block, and Li element—backward element. The numbers encode the angle (KBW only), step, and element length, in mm.

To prepare the second type of samples, a co-rotating, twin-screw extruder Bühler BTSK (Uzwil, Switzerland) with a screw diameter of 20 mm and L/D ratio of 40 was used. The screw system, shown in Figure 2, has a high mixing ability, and it includes four mixing and shearing zones. The first zone of the screw system consists of two kneading elements, KBW 90/2/15 and KBW 45/5/20. The next zones have forward kneading elements and an additional reverse kneading element KBW 45/5/20 Li. The reverse element holds the polymer in the zone for a longer period, resulting in more intense mixing, but this can lead to the degradation of PLA. All samples were extruded at the screw rotation speed of 100 rpm and temperature profile of 175 °C, 180 °C, 180 °C, 185 °C, and 185 °C (head).

The samples were extruded three times for each method, where each time a sample of the obtained composites was acquired for further analysis. The following parameters were registered during the extrusion process: changes of the temperature in each extruder zone, stock temperature, torque of the main drive, power of the main drive, and the process efficiency. The values of these parameters were recorded after they stabilized during extrusion. Obtained samples are listed in the Table 1.

### 2.3. Material Characterization

#### 2.3.1. Phase Morphology Analysis

Scanning electron microscopy (SEM, SU8010, Hitachi, Japan) was used to check the dispersion of MWCNT in the polymer matrix. 

For SEM imaging, samples were deposited on conductive carbon adhesive tape. Then, they were coated with a nanometric layer of gold, the presence of which increases the electrical conductivity of the sample surface. All microscopic observations were made at the accelerating voltage of 10 kV and a working distance of 8 mm.

#### 2.3.2. Spectroscope Analysis

XRD Diffractometer using Cu–Kα radiation (γ = 1.54056 Å, voltage 40 kV, current 30 mA), with a curved pyrdytic graphite crystal, fixed 1.0° divergence, fixed 1.0° anti-scatter, and fixed 0.1 mm receiving slits was used to perform X-ray diffraction analysis (XRD). The 2*θ*-angle was calibrated with copper. Before XRD analysis, samples were mounted on flat stainless steel plates with rectangular holes. The scanning was performed in 0.02° steps, using 120.0 s time in the range 2*θ* = 10−70° at ambient temperature.

The size of the MWCNT crystal structure was calculated from XRD results, using the Scherrer equation [11]:

$$D=\frac{K\gamma }{Bcos\theta }$$

The average d-spacing (*d*) value of the MWCNT in samples was calculated by using Bragg’s law [12]:

$$d=\frac{\gamma }{2sin\theta }$$

*K* is Scherrer constant, *D* is the mean size of the crystalline domains, *γ* is the X-ray wavelength, *B* is the line broadening at half the maximum intensity (FWHM), and *θ* is the Bragg angle. The value of the K constant was assumed to be 0.89 [13,14]. Calculations were performed using the X’Pert Data Viewer software (size/strain calculator) for the highest intensity peak (2 *θ* ≈ 26.5°). 

Fourier transform infrared spectroscopy–attenuated total reflectance spectra were measured using the Cary 630 FTIR-ATR spectrometer (Agilent Technologie, Santa Clara, CA, USA). Parameters used in measurements were: spectral range 400–4000 cm^−1^, spectral resolution <2 cm^−1^, and signal-to-noise ratio (1 min, RMS) > 30,000:1.

Raman spectra were recorded in backscattering geometry with a Senterra Raman microscope (Bruker Optik, Billerica, MA, USA), using a 2 mW laser beam with a wave-length of 532 nm as an excitation light source. Both the ATR-FTIR and Raman spectra were acquired at ambient temperatures.

#### 2.3.3. Thermal Behavior and Stability Analysis

METTLER TOLEDO DSC1 differential scanning calorimeter was used to record glass transition temperature (*T_g_*), crystallization temperature (*T_c_*), cold crystallization temperature (*T_cc_*), melting temperature (*T_m_*), crystallization enthalpy (Δ*H_c_*), cold crystallization enthalpy (Δ*H_cc_*), and melting enthalpy (Δ*H_m_*). Samples of approximately 5–7 mg, sealed in aluminum crucible, were used. 

DSC analysis was divided into five stages:

- First stage (Heating 1): the samples were heated at a constant rate of 10 °C/min from 0 °C to 300 °C.
- Second stage: an isothermal stage lasting 5 min.
- Third stage: the samples were cooled at a rate of 10 °C/min to 0 °C.
- Fourth stage: an isothermal stage lasting 5 min.
- Fifth stage (Heating 2): the samples were heated at a constant rate of 10 °C/min.

The following expression was used to evaluate the room temperature crystallinity, *X_c_*, of PLA [15]:

(1) $$X_{C}=\left(\frac{\Delta H_{m}-\Delta H_{cc}}{w\Delta H_{m}^{0}}\right)\cdot 100\%$$

where Δ*H*_m_ is the fusion enthalpy (J/g), Δ*H*_cc_ is the cold crystallization enthalpy (J/g), $\Delta H_{m}^{0}$ is the enthalpy of fusion of 100% crystalline PLA (93 J/g), and *w* is the fraction of the polymer in the composite materials. The experiment was performed in accordance with the ISO 11357-(1–3): 2016 standards [16]. The glass transition, crystallization, and melting temperatures were determined with accuracy of ±0.8 °C and enthalpy with ±0.5 J/g.

#### 2.3.4. Melt Flow Rate

The melt flow rate (MFR) of the composites was determined according to the PN-EN ISO 1133:2011 standard [17] with a Dynisco LMI 4003 capillary plastometer. The measurements were carried out under the piston loading of 2.16 kg at 190 °C for samples without MWCNT and 21.6 kg at 210 °C for samples with MWCNT. The temperature and piston load were increased due to the lack of observable flow of melted composites at lower measurement conditions. For all samples, melt time was 240 s. 

## 3. Results

### 3.1. Extrusion Process Analysis

Table 2 and Table 3 show the values of stock temperature (T_t_), torque main drive (M_o_), power main drive (W), and efficiency (Y) recorded during extrusion; Table 4 and Table 5 show the differences between set and extrusion temperatures in extruder heating zones.

For samples extruded with a single- and twin-screw extruder, a common relationship was observed between the changes in torque, process efficiency, and the number of extrusions. Subsequent extrusions caused a decrease in the torque and efficiency of the process. The reason for the change in torque may be a reduction in the viscosity of the polymer [18], which is closely related to polymer molecular weight as well as chain length [19]. High temperature and mechanical forces accompanying extrusion cause shortening of polymer chains, leading to its degradation [20]. The changes are more pronounced for the single-screw extruder. 

The same relationships (decrease in M_o_ and Y as a function of the number of extrusions) occur for samples with the addition of nanocarbon filler. However, the presence of the 25 wt.% of filler manifests itself in a significant increase in torque compared with unfilled composites. As in the case of unfilled composites, a decrease in the extruder torque accompanied the subsequent extrusions. Mixing the polymer matrix of higher viscosity with the addition of agglomerated MWCNT leads to higher values of shear stresses and, thus, requires higher energy, which translates into an increase in the registered torque [21]. Each subsequent extrusion of the same composite improves the dispersion of carbon nanotubes, reducing the content of agglomerates, manifested by reducing the extruder load.

Changes between the set and actual extruder temperature zones were observed. While the subsequent extrusion of the samples without the addition of MWCNT did not show much of a change, the addition of the filler caused the rise of extrusion temperature. The biggest increases were with the first extrusion in heating zone 3 for the single-screw extruder and zones 2 and 3 for the twin-screw extruder. These zones contain the mixing and shearing elements of the applied plasticizing systems. The changes may be caused by the presence of large agglomerates of carbon nanotubes and, thus, an increase in the extrusion resistance [22]. As the number of extrusion increases, the differences between the set and actual extrusion temperatures decrease. The decrease in temperature can account for both a reduction in the viscosity of the polymer and a reduction in the number of agglomerates in the composite. These observations can be applied to all samples with MWCNT obtained with the single- and the twin-screw extruders. 

### 3.2. Phase Morphology Analysis

**Figure 3:**
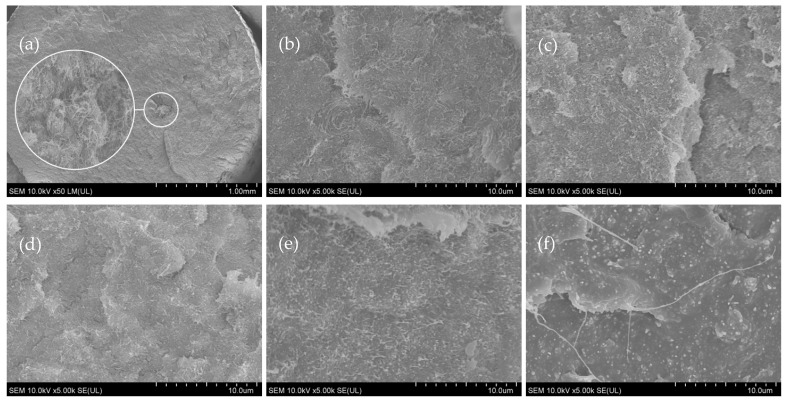
SEM images of PLA/MWCNT composite samples: (**a**) PLA/CNT/Sx1 (with MWCNT agglomerate), (**b**) PLA/CNT/Sx2, (**c**) PLA/CNT/Sx3, (**d**) PLA/CNT/Tx1, (**e**) PLA/CNT/Tx2, and (**f**) PLA/CNT/Tx3.

Figure 3 shows breakthrough of samples with carbon nanofillers (in the direction of extrusion). In the sample made with the use of a single-screw extruder, after a single extrusion, the presence of agglomerates of carbon nanotubes was noticed. They were not found in the two- and threefold extruded samples. In addition, in all the samples extruded with a twin-screw extruder, agglomerates were not observed.

No pores or any other types of discontinuity were found in the polymer matrix.

### 3.3. XRD Study

**Figure 4:**
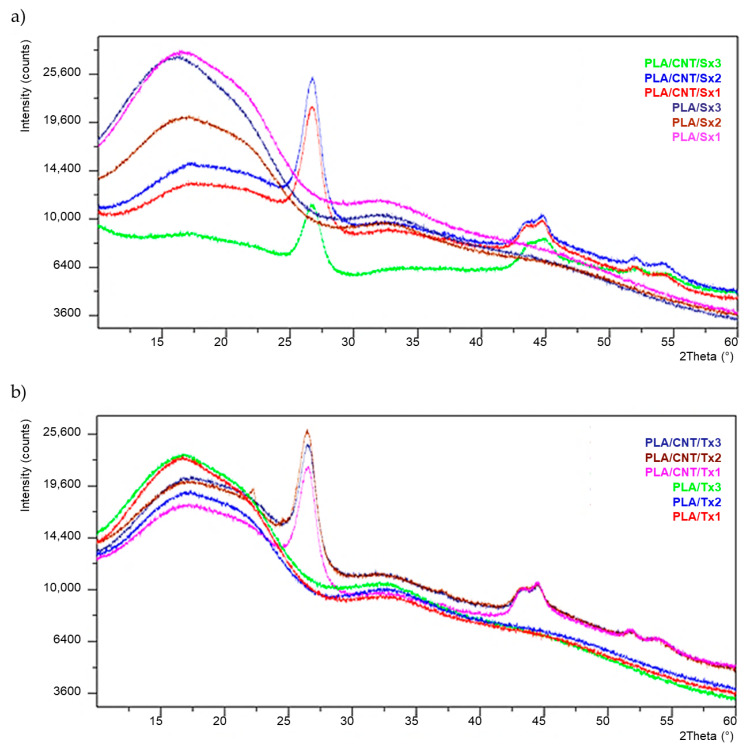
XRD patterns of neat PLA and PLA/CNT composites subjected to single-screw extruding (**a**) and twin-screw extruding (**b**).

Figure 4 shows XRD patterns of the neat PLA and PLA/CNT composites subjected to the extruding process. A wide halo in the range of 2 *θ* = 10–20° with a peak around 2 *θ* = 16° is visible. The lack of sharp peaks in this range proves that polylactide has an amorphous microstructure without crystalline regions [23]. The applied plasticizing system and the subsequent extrusion processing does not affect the degree of polymer crystallinity.

In the XRD patterns of PLA/MWCNTs, the sharper peaks corresponding to the MWCNTs are apparent. The peak at 2 *θ* = 26.5° is the diffraction peak of (002) plane corresponding to spacing between the adjacent graphene layers in the multilayer cylinder walls. Peaks at 2 *θ* = 43.5° and 2θ = 44.6°, correspond to (100) and (101) lattice constants within the layers, respectively [24]. The week peak at 2 *θ* = 51.9° corresponds to the reflection (004) from spacings between adjacent graphite layers [25].

The results presented in Table 6 were calculated for the most intense peak 2 *θ* ≈ 26.5°. No changes in the position of this peak were observed in samples differing in both the number of extrusions and the type of extruder used. Changes in the position of this peak (shifts to lower angles) may indicate an increased interaction between polylactide and carbon nanotubes [26].

The d-spacing value near 3.35 Å is characteristic of graphite layer structures [27]. An increase in the distance between the graphene layers indicates MWCNT exfoliation [28]. The D value corresponds to mean size of the crystalline domains. The lack of changes in these values proves that multiple extrusions have no effect on the structure of the multiwall carbon nanotubes used.

### 3.4. FTIR and Raman Analysis

**Figure 5:**
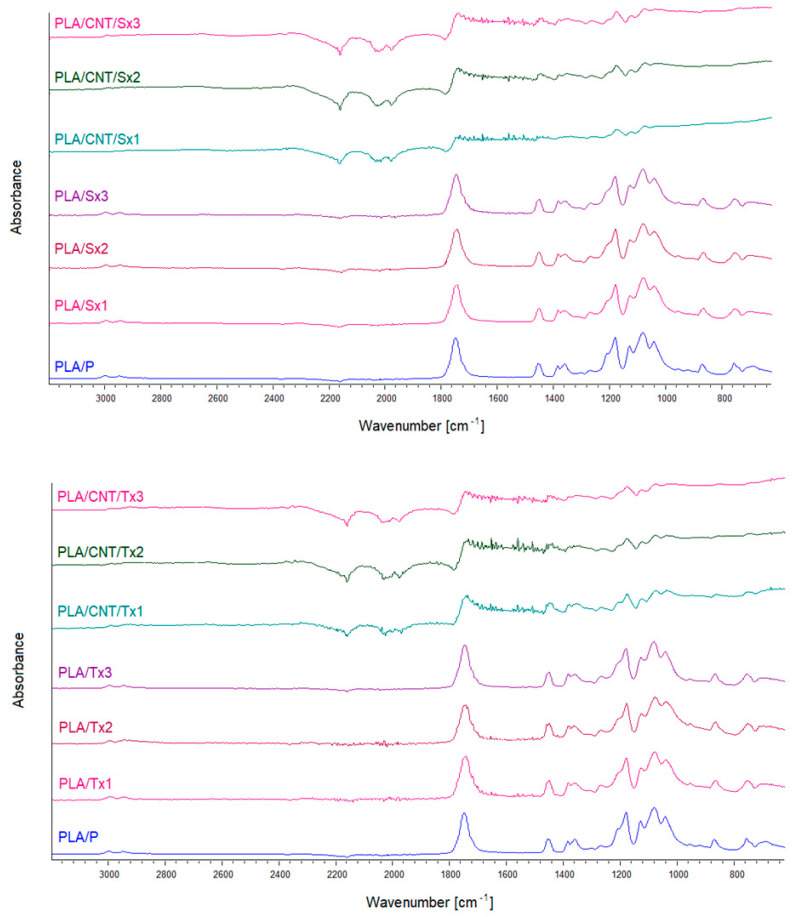
ATR-FTIR spectra of neat PLA and PLA/MWCNT composite subjected to the single, double, and triple extrusion. Upper panel shows spectra of samples subjected to the single-screw extrusion, bottom panel refers to the twin-screw extrusion. At the bottom of each panel, spectrum of the neat unprocessed PLA (PLA/P) is shown as the reference.

FTIR spectra of the neat polymer and PLA with MWCNT filler are shown in Figure 5. Spectra of the neat polymer contain the bands that are assigned to the PLA. Two bands at 755 and 870 cm^−1^ are attributed to the crystalline and amorphous phases, respectively, of the polymer [29]. At 1041 cm^−1^, stretching modes of the C-CH_3_ group appear. The symmetric and asymmetric stretching modes of the C-O-C group appear at 1081 (symm.) 1180 and 1266 cm^−1^ (asymm.), respectively. The rocking modes of the CH_3_ group are visible at 1129 cm^−1^. The CH and CH_3_ symmetric bending modes appear at 1357 and 1382 cm^−1^. The asymmetric bending modes of this group can be found at 1452 cm^−1^. Then, the C=O stretching modes of the ester group appear at 1747 cm^−1^. There are also weak bands at 2946 and 2994 cm^−1^ attributed to the asymmetric stretching modes of the CH_3_ group [30,31]. 

Subsequent extrusion processes do not change the intensity of the carbonyl (1747 cm^−1^) and ester (1081 cm^−1^ and 1180 cm^−1^) groups which accompany changes in the length of the polymer chains [32]. This means that the subsequent extrusions do not have a significant effect on the oxidating degradation of the polymer.

The characteristic bands assigned to PLA are also visible in samples with MWCNT. However, due to the strong carbon nanotube background absorption, the PLA bands are hardly visible [33].

**Figure 6:**
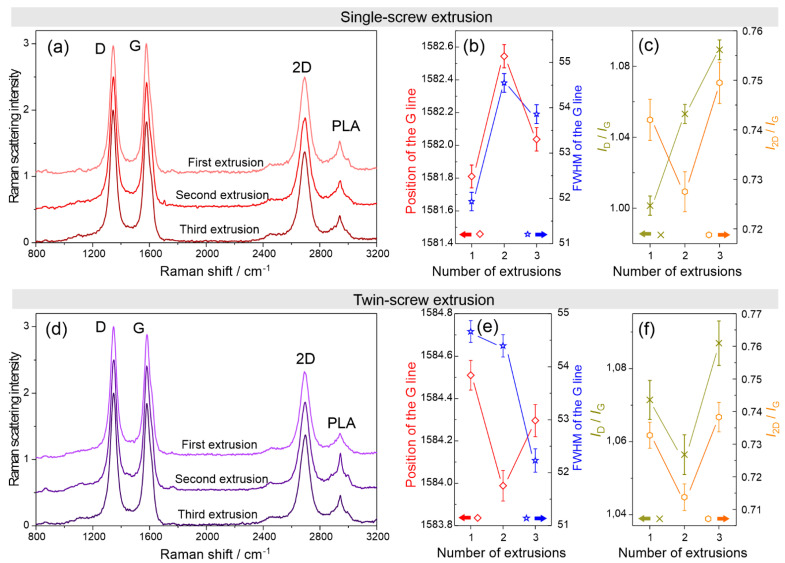
Raman spectroscopy of the PLA/MWCNT composites. (**a**) Raman spectra of PLA/MWCNT composites subjected to the single-screw extrusion. (**b**) Position and FHWM of the G line as a function of the number of single-screw extrusions. (**c**) Intensity of the D and 2D line against the intensity of the G line as a function of single-screw extrusions. (**d**) Raman spectra of PLA/MWCNT composites subjected to the twin-screw extrusion. (**e**) Position and FHWM of the G line as a function of the number of twin-screw extrusions. (**f**) Intensity of the D and 2D line against the intensity of the G line as a function of the twin-screw extrusions.

Influence of subsequent single- and twin-screw extrusions on the structure of MWCNT filler was investigated by Raman spectroscopy. In addition to strong G, D, and 2D sp^2^-bonded carbon features, a band at approximately 2940 cm^−1^ is visible in Raman spectra (Figure 6a,c); this is attributed to the stretching of the CH and asymmetric stretching of the CH_3_ group of PLA. Subsequent extrusions result in changes of the G line position of carbon nanotubes, its full width at half maximum (FHWM) and intensity ratios of the disorder-induced D line, its second order 2D-mode, and G band (*I*_D_/*I*_G_, *I*_2D_/*I*_G_). We observed essential differences between composites processed in single-screw and twin-screw extruders. Carbon nanotubes in composites subjected to single-screw extrusion show blue-shift by 0.7 cm^−1^ of the G-line, accompanied by broadening of the line after the second extrusion. The third extrusion results in slight red-shift (0.5 cm^−1^) and re-narrowing of the line (Figure 6b). To assess whether the changes in the G-line are caused by strain or by doping, we used the approach proposed by Mueller et al. [34]. 

The parameter describing the contribution of the strain and doping into the shift of the Raman G and 2D features is the Δω_2D_/Δω_G_ slope. In the case of the pure strain, Δω_2D_/Δω_G_ = 2.2. For the pure hole doping, Δω_2D_/Δω_G_ = 0.55. Pure electron doping can produce positive and negative slope coefficient values; however, its absolute value is always small compared with the stress-related value. Relative shifts, Δω_2D_/Δω_G_, caused by second and third single-screw extrusions are −0.24 and −0.44, respectively (data for the 2D line shift are not shown). Thus, we can conclude that subsequent processing in the single-screw extruder results in electron doping of carbon nanotubes in the PLA matrix. This statement is supported by a change in the line width that is correlated with the shift of the line position [35]. 

Different behavior is shown in the carbon nanotubes in the PLA matrix subjected to twin-screw extrusion (Figure 6e). The second processing results in the red-shift of the G line. A consequence of the next extrusion is a slight blue-shift of the G mode. Relative shifts, Δω_2D_/Δω_G_, caused by second and third extrusion are positive and reach values much higher than those which are predicted for the pure doping (1.48 and 0.96, respectively). Moreover, there is no correlation between the line shift and line width. From this behavior it can be concluded that the twin-extrusion causes strains in the fillers. 

Relative intensities of the D and 2D features against the G mode shown in Figure 6c and f provide information about defects and interactions between the sp^2^-bonded carbon tubular layers, respectively [27]. The defect-induced D-mode relative intensity increased after each single-screw extrusion, indicating the increased concentration of defects. On the other hand, the twin-screw extrusion results in the drop of the *I*_D_/*I*_G_ after the second extrusion. Reduction of defect concentration is likely caused by residual strain. The subsequent third twin-screw extrusion causes an increase of the *I*_D_/*I*_G_ in both the single- and twin-screw extrusion.

The changes of the *I*_2D_/*I*_G_ ratio are similar in the MWCNT/PLA composite subjected to single- and twin-screw extrusion. On the basis of this, a cautious conclusion can be drawn that the second extrusion leads to increased interactions between the nanotubes in the polymer matrix (aggregation), while the subsequent treatment improves the dispersion of MWCNT filler.

### 3.5. Thermal Behavior and Stability Analysis

**Figure 7:**
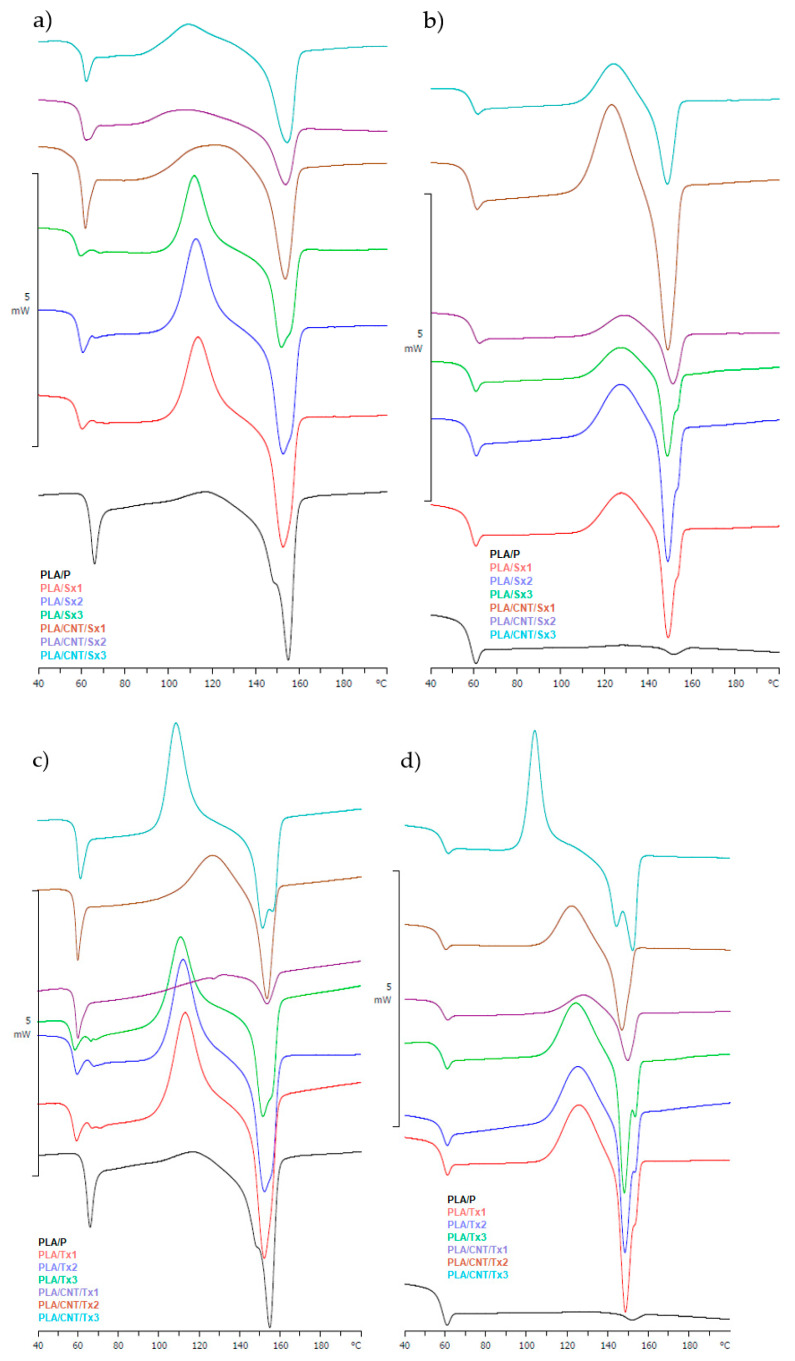
DSC analyses of composites produced by: single-screw extruder: (**a**) first heating and (**b**) second heating; twin-screw extruder: (**c**) first heating and (**d**) second heating.

Figure 7 shows the DSC thermograms of the obtained samples. Thermal data, such as the glass transition temperature (*T_g_*), crystallization temperature (*T_c_*), cold crystallization temperature (*T_cc_*), melting temperature (*T_m_*), crystallization enthalpy (Δ*H_c_*), cold crystallization enthalpy (Δ*H_cc_*), and melting enthalpy (Δ*H_m_*) are summarized in Table 7. The heating scan of the pristine PLA (sample PLA/P) showed an endothermic peak corresponding to the melting of the polymer (T_m_^1^ = 154.8 °C). This peak was not observed in the second heating scan. PLA is a polymer that crystallizes slowly. The applied sample cooling rate (10 °C/min), before the second heating, did not allow the formation of a crystalline phase in the molten polymer [35]. This speed is too fast for the polylactide chains to reorganise into crystal regions [36]. The absence/large reduction of melting peak in Heating 2 has also been reported by other researchers [37,38,39].

During the first heating. the endothermic glass transition of PLA occurred at approximately 56–64 °C. At this temperature, the polymer chains are able to move. The temperature of this transition is approximately 2 °C higher for samples containing nanocarbon filler compared with non-filled composites. The increase in temperature is related to the restriction of the mobility of the polymer chains by MWCNT [40]. No relationships were observed among the number of extrusions, type of extruder, or the glass transition temperature. 

Reorganization of amorphous domains into crystalline domains manifests as an exothermic peak at 110–113 °C in the case of composites without filler and 108–131 °C for PLA/MWCNT composites. As in the case of the glass transition, the increase in *T_cc_* is related to the restriction of the mobility of the polymer by the filler [40].

Differences in cold crystallization enthalpy, in Heating 1, are clearly seen. The Δ*H_cc_* of samples containing MWCNT is lower than that for non-filled samples. The observed differences can be explained by the quality of the crystallites formed during cold crystallization. The presence of carbon nanotubes in the composite limits the space in which crystals could form. The result is the growth of small, imperfect crystallites [41]. The increase in the enthalpy of cold crystallization with each successive extrusion is related to the increasingly better dispersion (decrease in the amount of agglomerates) of the nanofiller in the sample [42]. 

The melting point *T*_m_ of all samples is comparable. The melting enthalpy Δ*H*_m_ of the sample without MWCNT is similar to the neat PLA sample and approximately 14–25 J/g higher than the melting enthalpy of the nanocarbon-containing samples. Since the degree of crystallinity of samples containing nanotubes and samples without it is at a similar level, it can be assumed that the quality of the formed crystallites is responsible for the change of the melting enthalpy [43]. 

During cooling, no effect related to polymer crystallization was observed for any of the analysed samples. 

The room temperature crystallinity X_c_ in the pure polymer decreased after each heating cycle. The X_c_ estimated from the first heating curve was found to be 36%. After the melting process (sample PLA/P), the *X_c_* decreased to 0%. The second heating caused the crystallinity to drop to almost zero in both the PLA/MWCNT and non-filled samples. The amorphous character of the obtained samples is also confirmed by analysis of XRD spectra.

During the second heating, the difference in glass transition temperature between filled and unfilled samples is not noticeable.

In Heating 2, a relationship among the number of extrusions of the samples, its temperature, and the cold crystallization enthalpy occurs for samples with MWCNT. Each subsequent extrusion resulted in a decrease in the values of *T_cc_* and an increase in Δ*H_cc_*. As revealed by molecular simulations, local mobility of the polymer chains near the carbon filler phase are highly anisotropic and drastically reduced. Thus, carbon fillers can act as a nucleating agent that promotes the crystallisation process [8]. As a consequence, the *T_cc_* decreases. The decrease in this value with the increase in the number of sample extrusions may be related to the increasingly better dispersion of the filler in the polymer matrix. This phenomenon is more visible for the samples obtained with the twin-screw extruder.

### 3.6. Melt Flow Rate

Table 8 summarizes the melt flow rate results for individual samples. In the polymer processing industry, MFR is important to analyse the flow property of polymers since it is easy to measure.

The MFR measurements were carried out under different conditions for the samples with and without the addition of MWCNT. Increasing the piston load (×10) and temperature (by 20 °C) was due to the fact that there was no running flow across the capillary at the standard measurement conditions (2.16 kg, 190 °C). The addition of 25 wt.% of multi-wall carbon nanotubes increases the flow resistance of the molten polymer associated with the use of solid filler [44]. 

An increase in MFR value may be a sign of degradation of the polymers [45]. The influence of high temperature and shear forces acting on the polymer chains during extrusion causes its mechanical shortening (chain breaking), which can reduce the viscosity of material and increase its flow [20]. Increase in the value of this parameter was observed for samples without filler with increased number of extrusions. This same observation can be made for samples with MWCNT. However, increase of MFR for samples made with twin-screw extruders is much higher than for single-screw extruders. 

## 4. Conclusions

In conclusion, we show that multiple extrusions strongly affect the processing parameters. The introduction of nanotubes into the die causes an almost twofold reduction in extrusion efficiency. At the same time, the power consumption of the extruder increases. This interdependence is more visible in the single-screw extruder. Subsequent extrusions result in decreased torque and process efficiency.

SEM imaging combined with XRD and Raman studies show differences between properties of PLA/MWCNT composites obtained using single- and twin-screw extrusion. We show that single-screw extrusion is less efficient than the twin-screw extrusion. No effects connected to the residual strain are observed in PLA/MWCNT subjected to the single-screw extrusion, whereas they are apparent in the samples subjected to the twin-screw extrusion. 

As revealed by DSC analyses, cold crystallization temperature shows a tendency to decrease after each process, whereas cold crystallization enthalpy ΔH_cc_ increases significantly. This proves that the PLA crystallization process, during DSC analysis, is supported by the presence of the MWCNT filler in obtained composites. However, all samples have an amorphous character, which is proved by the results of the XRD analysis. The cooling rate of the extruded composite is too high for the polymer crystallization process to occur.

During extrusion, the polymer undergoes partial degradation, as evidenced by the increase in the melt flow rate. Since the FTIR-ATR spectra of the obtained samples showed no differences between them and virgin PLA, it can be assumed that the degradation occurs by mechanical shortening of the polymer chains without the formation of carbonyl structures. 

The addition of carbon nanotubes to PLA causes a significant increase in MFR. Composites with this addition did not show flow under standard measurement conditions for polylactide. This increase is related to the presence of a solid filler in the composite and likely to the geometry of the nanotubes themselves. It should be emphasized that the increase in MFR occurs more quickly when PLA/CNT mixtures are processed with a twin-screw extruder.

The extrusion process using a single-screw extruder is less efficient and requires more torque than a twin-screw extruder. However, this leads to obtaining composites with much lower values of the mass flow rate. The use of a single-screw extruder requires at least one repetition of the extrusion process in order to eliminate the presence of agglomerates in the obtained composites. A good dispersion of carbon nanotubes in the polymer matrix can be obtained after a single extrusion using a twin-screw extruder. Properties of samples subjected to the double extrusion on single-screw extruders are comparable to samples subjected to single twin-screw.

The knowledge gained during the preparation of this publication will be used in future experiments aimed at testing polymer composites highly filled with nanomaterials in terms of, among others, electrical properties, thermal conductivity, and mechanical strength. The obtained composites can be used as polymer masterbatches in future works.

## Figures and Tables

**Table 1:** List of abbreviations and formulations of the tested PLA/MWCNT composites.

Code	MWCNT (wt.%)	Single/Twin-Screw Extruder	Number of Extrusion Process
PLA/P	0	virgin	
PLA/Sx1	0	single	1
PLA/Sx2	0	single	2
PLA/Sx3	0	single	3
PLA/CNT/Sx1	25	single	1
PLA/CNT/Sx2	25	single	2
PLA/CNT/Sx3	25	single	3
PLA/Tx1	0	twin	1
PLA/Tx2	0	twin	2
PLA/Tx3	0	twin	3
PLA/CNT/Tx1	25	twin	1
PLA/CNT/Tx2	25	twin	2
PLA/CNT/Tx3	25	twin	3

**Table 2:** Parameters recorded during extrusion—single-screw extruder.

Single-Screw Extruder						
Sample	PLA/Sx1	PLA/Sx2	PLA/Sx3	PLA/CNT/Sx1	PLA/CNT/Sx2	PLA/CNT/Sx3
M_o_ [Nm]	41.0	34.0	24.0	60.0	54.0	50.0
Y [kg/h]	2.70	2.18	1.57	1.12	0.98	0.90

**Table 3:** Parameters recorded during extrusion—twin-screw extruder.

Twin-Screw Extruder						
Sample	PLA/Tx1	PLA/Tx2	PLA/Tx3	PLA/CNT/Tx1	PLA/CNT/Tx2	PLA/CNT/Tx3
Tt [°C]	198	200	200	207	203	198
M_o_ [Nm]	21.1	20.1	18.5	29.7	28.1	26.4
W [kW]	0.38	0.39	0.36	0.59	0.52	0.49
Y [kg/h]	3.25	3.10	3.05	1.85	1.83	1.80

**Table 4:** Set and extrusion temperature—single-screw extruder.

Single-Screw Extruder				
Sample	Zone 1 [°C]	Zone 2 [°C]	Zone 3 [°C]	Head [°C]
Set temperature	170	175	180	180
PLA/Sx1	170	175	180	180
PLA/Sx2	172	174	179	180
PLA/Sx3	170	175	180	180
PLA/CNT/Sx1	170	201	210	180
PLA/CNT/Sx2	172	184	187	180
PLA/CNT/Sx3	172	183	186	179

**Table 5:** Set and extrusion temperature—twin-screw extruder.

	Twin-Screw Extruder				
Sample	Zone 1 [°C]	Zone 2 [°C]	Zone 3 [°C]	Zone 4 [°C]	Head [°C]
Set temperature	175	180	180	185	185
PLA/Tx1	176	183	183	185	183
PLA/Tx2	172	178	181	186	182
PLA/Tx3	170	179	180	185	186
PLA/CNT/Tx1	177	200	196	188	188
PLA/CNT/Tx2	179	190	182	187	185
PLA/CNT/Tx3	177	185	186	188	183

**Table 6:** XRD analysis results.

Sample	Peak Position 2*θ* [°]	FWHM [°]	d-Spacing [Å]	*D* [nm]
PLA/CNT/Sx1	26.72	1.38	3.33	5.99
PLA/CNT/Sx2	26.71	1.35	3.33	5.86
PLA/CNT/Sx3	26.73	1.49	3.33	5.42
PLA/CNT/Tx1	26.49	1.27	3.36	6.36
PLA/CNT/Tx2	26.46	1.29	3.37	6.26
PLA/CNT/Tx3	26.51	1.26	3.36	6.41

**Table 7:** Thermal data obtained by DSC.

Sample	Heating 1						Heating 2					
	T_m_ [°C]	∆H_m_ [J/g]	T_g_ [°C]	T_cc_ [°C]	ΔH_cc_ [J/g]	X_c_^1^ [%]	T_m_ [°C]	∆H_m_ [J/g]	T_g_ [°C]	T_cc_ [°C]	ΔH_cc_ [J/g]	X_c_^2^ [%]
PLA/P	154.8	28.0	64.1	116.6	5.0	35.5	-	-	58.5	-	-	0
PLA/Sx1	152.5	27.9	57.8	113.3	27.9	0	149.2	13.9	58.2	127.6	13.8	0
PLA/Sx2	152.6	27.0	58.3	112.4	27.0	0	149.1	15.4	58.6	127.2	15.6	0
PLA/Sx3	151.2	29.8	57.0	111.6	27.1	2.1	148.8	13.0	58.5	127.0	12.5	0
PLA/CNT/Sx1	153.5	11.4	59.8	108.8	10.9	0	151.3	6.5	59.4	129.0	6.5	0
PLA/CNT/Sx2	153.5	11.8	59.9	119.3	11.1	0	149.1	15.0	58.6	123.1	14.7	0
PLA/CNT/Sx3	154.4	19.0	60.5	109.2	15.6	3.7	148.8	11.5	58.2	124.0	11.2	0
PLA/Tx1	152.2	27.2	56.7	112.9	26.8	0	148.5	20.6	58.7	125.7	20.5	0
PLA/Tx2	152.1	27.2	57.5	111.7	27.2	0	148.2	19.5	59.0	124.9	19.2	0
PLA/Tx3	151.4	27.9	56.4	110.5	27.5	0	147.9	23.0	58.8	124.2	22.2	0
PLA/CNT/Tx1	153.7	5.5	58.5	130.8	4.4	0	149.6	9.1	58.6	127.9	9.1	0
PLA/CNT/Tx2	153.4	16.3	58.5	126.2	16.3	0	146.8	14.6	57.1	122.2	14.5	0
PLA/CNT/Tx3	151.4 156.2	28.7	59.6	108.4	28.2	0	144.1 152.1	27.1	58.4	103.9	27.1	0

**Table 8:** MFR results for individual samples.

Sample	MFR (g/10 min)
**Piston load: 2.16 kg; melt temperature: 190 °C**	
PLA/P	7.81 ± 0.08
PLA/Sx1	7.47 ± 0.07
PLA/Sx2	10.69 ± 0.19
PLA/Sx3	13.36 ± 0.31
PLA/Tx1	7.62 ± 0.15
PLA/Tx2	9.01 ± 0.11
PLA/Tx3	11.17 ± 0.17
**Piston load: 21.6 kg; melt temperature: 210 °C**	
PLA/CNT/Sx1	0.07 ± 0.01
PLA/CNT/Sx2	0.80 ± 0.05
PLA/CNT/Sx3	1.06 ± 0.07
PLA/CNT/Tx1	40.00 ± 4.88
PLA/CNT/Tx2	105.10 ± 1.85
PLA/CNT/Tx3	174.30 ± 1.89

## Data Availability

The data presented in this study are available on request from the corresponding author.
